# Big data, medicines safety and pharmacovigilance

**DOI:** 10.1186/s40545-021-00329-4

**Published:** 2021-06-02

**Authors:** Rabia Hussain

**Affiliations:** Faculty of Pharmacy, The University of Lahore, Lahore, 54000 Pakistan

**Keywords:** Big data, Medicines safety, Pharmacovigilance, Artificial intelligence, Social media, COVID-19

## Background

Since the 1990s, the concept of big data has emerged as more relevant, diverse and larger data sets, responsible for the introduction of new drug developments, improved clinical practices and healthcare financing in the healthcare industry [1]. For big data analysis, one can handle a large pool of digital medical records or administrative data including drug safety reports, drug prescriptions as well as hospital discharge datasets [2].

Many rare adverse effects remain undetected due to a limited number of sampled individuals in a clinical trial; hence, it is necessary to monitor the drugs even after their release into the market. In this context, “pharmacovigilance” helps to collect, analyze, and disseminate adverse drug reaction reports collected during the post-marketing phase [3, 4].

**Fig. 1:**
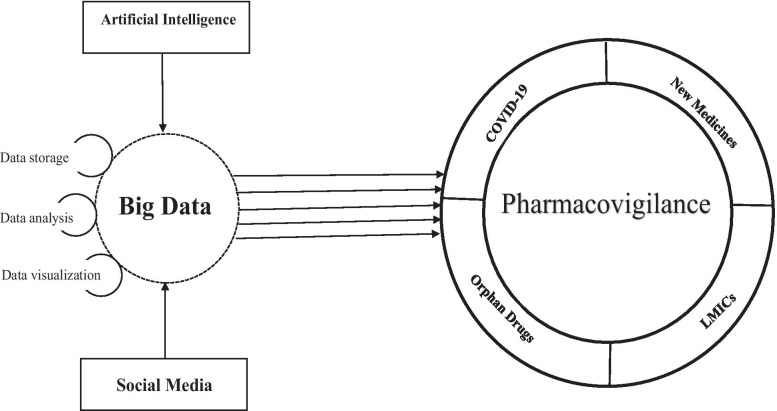
A framework showing the possible linkages between big data and pharmacovigilance

Data mining from drug safety report databases and medical literature is a time-consuming task; however, with the digital revolution, the researchers are exploring if the potential of big data could be used to study and monitor drug safety. In many developed countries, drug safety surveillance based on databases through automation is becoming increasingly common [2]. This involves the usage of electronic methods to systematically analyze the large volume of information. This could be further helpful to detect data patterns to identify new adverse drug reactions, which are otherwise not available through normal screening [2]. This commentary discusses big data, artificial intelligence and the use of social media. It also elaborates, how “big data” feeds into evaluating the safety of new and orphan medicines (Fig. 1).

## Artificial intelligence and pharmacovigilance

To better understand the use of artificial intelligence in pharmacovigilance, it may be useful to define this in terms of methods, tasks and data sets [5]. Machine learning is part of artificial intelligence that deals with the ability of machines to learn without having human input. Due to improved computational techniques and the availability of larger datasets, there is an increasing trend in machine learning adoption in healthcare [6].

For an automated signal generation in pharmacovigilance, both supervised and unsupervised machine learning approaches are used. The unsupervised machine learning approach employs the identification of drug safety signals as well as explores the pattern of drug utilization. While in supervised machine learning, the computer is provided with a set of instructions to produce an algorithm based on the desired output [7]. It could be explained by considering the identification of an ADR from free text [8]. This is done by creating an identification pattern extracting information from the medical records and then applying the algorithms to the full electronic medication records. The process is called natural language processing (NLP). It can be applied to identify drug interactions from clinical notes and to find the association between drugs and potential ADRs [9].

## Social media

With the increasing use of social media, it is becoming a very useful tool to promote pharmacovigilance. However, several regulatory and technical challenges need to be addressed before the true potential of social media could be explored. The data can be collected from either Twitter or Facebook where several patients share their personal experiences regarding a particular drug or therapy, thus providing a good source for early signal detection [10]. However, the challenge is the accuracy of the information being posted on these websites. Several methods are in place to cross-check the reliability of data. One such tool is to adapt "Fuzzy Formal Concept Analysis" as it verifies the data by checking the information with the official information sources [11]. Social media could be very useful particularly in low- and middle-income (LMICs) countries where it is difficult to obtain accurate electronic data and large populations have started using Twitter and Facebook.

## Orphan drugs

In the past, the treatment for rare diseases was a challenge. There was little interest to develop new medicines for these diseases due to little market incentives. To overcome this problem, in the United States, several initiatives were taken including the United States drug act of 1983, the Rare Diseases Act of 2002, the Precision Medicine Initiative, and the 2016 Orphan Products Natural History Grants Program. As a result, the number of orphan medicines increased, and by 2016, 3735 products were registered as orphan drugs in the US. Also, 1314 medicines were registered in Europe [12]. The number of people who are using orphan drugs is very small, hence conducting pharmacovigilance is a challenge. However, to solve these issues, in some countries, patient support programs (PSPs) are established. The purpose is to create awareness about orphan diseases and medicines use. It is expected that these programs may help to produce drug safety reports too [13].

## COVID-19 and pharmacovigilance

In the era of COVID-19, medicines usage and pharmacovigilance are transforming rapidly, and a large volume of data is generated. The analysis of such a big volume of data requires both the involvement of artificial intelligence and big data analytical techniques. This is also creating opportunities for researchers and healthcare professionals to map innovative solutions during COVID times [14–16]. The pandemic is also resulting in large investments and the focus is on much needed infrastructure to monitor vaccine safety. It has also resulted in renewed interest in this area in a spectrum of middle and high-income countries.

## Conclusion

This commentary sets out the scene with regard to big data, medicines safety, and pharmacovigilance. It narrates how access to big data can improve medicines' safety. The framework describes the influence of social media and artificial intelligence on big data analytics. It also explained how this feeds into evaluating orphan and new medicines especially vaccines in the COVID-19 context.

## Abbreviations

US: United States
NLP: Natural language processing
PSP: Patient support programs
LMICs: Low- and middle-income countries
